# Extensive sequence-influenced DNA methylation polymorphism in the human genome

**DOI:** 10.1186/1756-8935-3-11

**Published:** 2010-05-24

**Authors:** Asaf Hellman, Andrew Chess

**Affiliations:** 1 Department of Developmental Biology and Cancer Research, Institute for Medical Research Israel-Canada, The Hebrew University-Hadassah Medical School, Jerusalem 91120, Israel; 2 Center for Human Genetic Research and Department of Medicine, Massachusetts General Hospital, Harvard Medical School, 185 Cambridge St., Boston, Massachusetts 02114, USA; 3 Broad Institute, Cambridge, Massachusetts, USA

## Abstract

**Background:**

Epigenetic polymorphisms are a potential source of human diversity, but their frequency and relationship to genetic polymorphisms are unclear. DNA methylation, an epigenetic mark that is a covalent modification of the DNA itself, plays an important role in the regulation of gene expression. Most studies of DNA methylation in mammalian cells have focused on CpG methylation present in CpG islands (areas of concentrated CpGs often found near promoters), but there are also interesting patterns of CpG methylation found outside of CpG islands.

**Results:**

We compared DNA methylation patterns on both alleles between many pairs (and larger groups) of related and unrelated individuals. Direct observation and simulation experiments revealed that around 10% of common single nucleotide polymorphisms (SNPs) reside in regions with differences in the propensity for local DNA methylation between the two alleles. We further showed that for the most common form of SNP, a polymorphism at a CpG dinucleotide, the presence of the CpG at the SNP positively affected local DNA methylation in *cis*.

**Conclusions:**

Taken together with the known effect of DNA methylation on mutation rate, our results suggest an interesting interdependence between genetics and epigenetics underlying diversity in the human genome.

## Background

In theory, DNA methylation patterns can be transmitted directly between human generations if the methylation (or other epigenetic mark that can be a placeholder) is maintained during gametogenesis and embryonic development. Another possible mode of transmission is through DNA sequence polymorphisms that affect methylation prevalence at linked sites. However, the nature of polymorphic sequences affecting methylation is not clear. Mendelian inheritance of methylation patterns near variable number tandem repeats (VNTRs) has been reported [1]. Three of the 10 analyzed VNTRs had evidence of a heritable methylation pattern, suggesting this may be a relatively widespread phenomenon across the genome. In the case of these VNTR-associated methylation differences, it was not determined whether the methylation differences were sequence-independent, whether sequences linked to the VNTRs were involved, or whether particular aspects of the repeats themselves were somehow influencing nearby methylation. The idea that this might be a prevalent mechanism came with the caveat that the genomic regions analyzed were small in number, and were all VNTRs. More recent analyses of a potential connection between single nucleotide polymorphisms (SNPs) and DNA methylation using genotyping arrays came to a different conclusion: that regions of the genome in which DNA sequence differences influence local DNA methylation are rather rare. One study arrived at a lower limit of 0.16% of SNPs [2], and another study suggested that 1.5% of SNPs could affect local methylation [3]. Thus, the extent to which SNPs may direct methylation remains unclear. Other studies in humans have focused on an imprinted locus [4,5] or examined CpG islands [6], and a recent study on F1 mice focused on regions preselected to have strain-specific differences in gene expression [7]. We report our analyses of DNA methylation at over 100,000 CpGs distributed throughout the genome, and our investigation into the dependence of DNA methylation on nearby sequence polymorphisms.

## Results and Discussion

**Figure 1:**
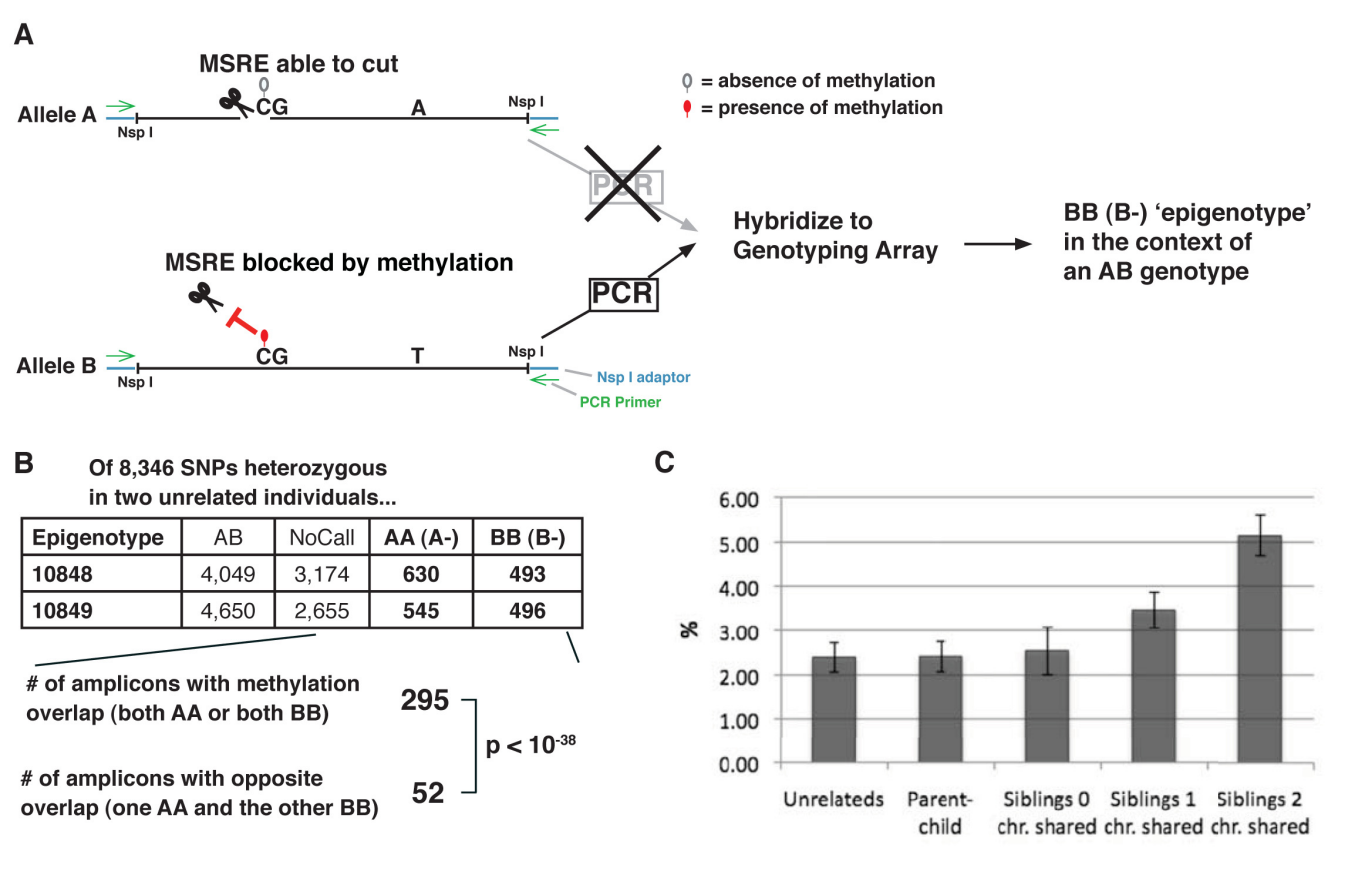
**Genome-scale assessment of allelic DNA methylation patterns**. **(a) **Representative amplicon (defined by *Nsp*I sites). The 'B' allele has a methylated CpG (red filled oval) within a methylation-sensitive restriction enzyme (MSRE). The 'A' allele is unmethylated at the CpG within the MSRE site, and is therefore digested upon the treatment with the MSRE cocktail that is performed before *NSP*I digestion ligation of adaptors, PCR amplification and hybridization to the Affymetrix 250 K single nucleotide polymorphism (SNP) mapping array. Only the 'B' allele yields a PCR product that can hybridize on the SNP-detecting array. **(b) **Of the 8,346 SNPs heterozygous in two genetically unrelated individuals (GM10848 and GM10849), epigenotypes are shown, with the numbers of amplicons with monoallelic methylation in bold. The lower part of the panel shows the number of amplicons displaying methylation overlap and the number displaying opposite overlap. The *P *value was calculated using the χ^2 ^test; the null hypothesis was equality in the numbers of overlapping SNPs with the opposite or same epigenotype. **(c) **The observed methylation overlap frequencies in excess of the levels expected by chance are presented for various types of comparisons of pairs of individuals.

To explore the potential effect of polymorphisms on local DNA methylation, we performed numerous comparisons of monoallelic methylation patterns in pairs of related and unrelated individuals. Methylation of the two alleles was measured using a SNP mapping array (Human Mapping 250 K array; Affymetrix, Santa Clara, CA, USA), with pre-digestion of the DNA with a cocktail of methylation-sensitive restriction endonucleases (MSREs) before PCR amplification of amplicons ranging in size from 100, to 1,100 bp (average 677 bp) [8] and subsequent detection of SNPs present on the amplicons. After all known MSRE site polymorphisms were filtered out, 110,883 amplicons were available for assessment of DNA methylation. As shown in Figure 1a, a heterozygous SNP present on an amplicon that has methylation present on only one of the two alleles will lead to a 'homozygous' call after MSRE treatment. Therefore, by looking for cases in which the genotype is heterozygous (in regular genomic DNA) and MSRE-pretreated DNA yields a homozygous call, amplicons with monoallelic methylation can be identified.

The first type of analysis measured the frequency of monoallelic methylation per person, for 13 individuals of different ages and gender. Focusing on amplicons containing a heterozygous SNP and at least one MSRE site, we determined that an average of 20% of the assessable amplicons had monoallelic methylation see Additional file 1. We examined the distribution of these cases of monoallelic methylation across the genome and found no evidence for clustering, either in individual chromosomes see Additional file 2 or in 1 Mb windows along the genome. Locus-specific validation was performed using bisulfite sequencing of 25 methylation sites see Additional file 3. What these analyses showed was that there were differences in methylation, detectable in amplicons heterozygous for a common SNP. Although the presence of the SNP was essential to our ability to observe monoallelic methylation, this method could not determine whether the observed monoallelic methylation was influenced by the SNPs or by other sequence differences in linkage disequilibrium (LD) with the SNPs.

### Assessing the effect of SNPs on local DNA methylation in unrelated individuals

We next analyzed the extent to which monoallelic methylation across the genome was influenced by local sequence differences between the two alleles. One way to examine the potential effects of sequence differences on allele-specific methylation involved the examination of informative amplicons in pairs of individuals; the SNP within each informative amplicon had to be heterozygous in each of two unrelated individuals and the amplicon had to contain at least one MSRE site. The null hypothesis would be that sequence differences have little or no effect on allelic methylation patterns. Under the null hypothesis, when examining informative amplicons that are heterozygous in each of two unrelated individuals, the frequency of amplicons displaying monoallelic methylation on the same alleles in both individuals (methylation overlap) would be equal to the frequency of amplicons displaying monoallelic methylation on different alleles (opposite overlap).

For example, consider the pair of unrelated individuals shown in Figure 1b. Of 8,346 informative SNPs, 295 (3.5%) had methylation overlap and 52 (0.6%) had opposite overlap (*P *< 10^-38^), thus the ratio of methylation overlap to opposite overlap is 5.7 for this pair. When DNA methylation was analyzed in 14 pairs of genetically unrelated individuals, amplicons showing methylation overlap were, on average 5.2-fold more frequent than amplicons showing opposite overlap (range 2.5-fold to 9.3-fold, with *P *values ranging from 10^-7 ^to 10^-66^; Table 1). This bias can be explained if many of the assessed regions are subject to allelic methylation that is influenced by the sequence differences between the two alleles.

**Table 1:** Analyses of pairs of unrelated individuals

								Observed		MO/OO	Expected			
Ind. 1	Ind. 2	Cell Type	I - SNPs	Ind. 1 A-	Ind. 1 B-	Ind. 2 A-	Ind. 2 B-						*P*	
								MO	OO		MO	OO		MO: (O–E)/I, %
GM12096	GM10849	LBL	7525	440	379	421	417	207	38	5.4	45.6	46.5	3.6 × 10^-27^	2.14

GM12097	GM10849	LBL	5469	663	590	315	261	172	30	5.7	66.3	65.6	1.7 × 10^-23^	1.93

GM12099	GM10848	LBL	9266	761	630	839	649	418	45	9.3	113.0	110.3	2.6 × 10^-67^	3.29

GM10848	GM10849	LBL	8346	630	493	545	496	295	52	5.7	70.4	69.6	6.8 × 10^-39^	2.69

GM10848	GM12698	LBL	8189	665	533	387	320	217	40	5.4	52.3	51.2	2.4 × 10^-28^	2.01

GM10848	GM12699	LBL	7678	575	491	710	578	323	46	7.0	90.1	88.7	3.9 × 10^-47^	3.03

GM10848	GM12706	LBL	9944	802	674	1234	1179	461	124	3.7	179.4	178.7	4.0 × 10^-44^	2.83

GM10849	GM12698	LBL	7340	483	414	315	248	147	32	4.6	34.7	34.1	8.3 × 10^-18^	1.53

GM10849	GM12699	LBL	7141	461	392	641	548	290	39	7.4	71.5	70.6	1.5 × 10^-43^	3.06

GM10849	GM12706	LBL	9118	636	537	1044	1071	356	112	3.2	135.9	136.2	1.7 × 10^-29^	2.41

GM12698	GM12699	LBL	7013	310	269	644	536	167	43	3.9	49.0	48.4	1.2 × 10^-17^	1.68

GM12089	GM12706	LBL	9627	1136	859	1142	1116	439	173	2.5	234.3	233.6	5.8 × 10^-27^	2.13

PWBC1	PWBC3	WBC	6713	155	203	102	147	69	15	4.6	6.8	6.5	3.8 × 10^-09^	0.93

PWBC2	PWBC3	WBC	7405	101	138	124	174	67	16	4.2	4.9	4.7	2.2 × 10^-08^	0.84

Averages										5.2				2.18

I = informative; Ind. = individual; MO = methylation overlap; O E = observed minus expected; OO = opposite overlap; SNP = single nucleotide polymorphism.

For the comparison of MO versus OO, the χ^2 ^*P *values were calculated.

Another way to examine the data from pairs of unrelated individuals is to examine the observed frequency of methylation overlap compared with the frequency that would be expected by chance (given the extent of monoallelic methylation in each of the two individuals who make up the pair). Under the null hypothesis - if monoallelic methylation were not influenced by sequence differences - the frequency of methylation overlap would be expected to equal the product of the frequencies of monoallelic methylation of the 'A' alleles in each individual, plus the same product for the 'B' alleles. For the example presented in Figure 1b, this would translate as (630/8346) × (545/8346) + (493/8346) × (496/8346) = 0.8% expected. However, the observed methylation overlap for this pair was 3.5%, thus giving an excess (over that expected by chance) methylation overlap of 2.7%. Examining methylation overlap in the 14 pairs of individuals, the average frequency of methylation overlap in excess of that expected by chance was 2.2% (Table 1; Figure 1c). Moreover, because different amplicons are informative in different pairs of individuals, the total fraction of amplicons (across the genome) that are associated with differences in the propensity for local methylation must be substantially higher (see below).

Because these individuals were unrelated and therefore only shared the SNPs measured by the array (and local common sequence polymorphisms in LD with these SNPs), these analyses highlighted a role for sequence polymorphisms common to the whole population in marking local differences in methylation propensity. Note that we also observed significant methylation overlap in pairwise comparisons of a variety of (freshly isolated) non-hematopoietic tissues from unrelated individuals see Additional file 4, suggesting that these allelic methylation patterns are present from early in development. Parental imprinting [9] was shown not to be a major contributor to the observed methylation overlap (see Additional files 5 and 6).

### Increased similarity in allelic methylation patterns in related individuals

Up to this point, the analyses of unrelated individuals presented suggested an influence of common allelic sequence differences on local methylation. This led to the question of whether the increased sequence identity in related individuals would lead to increased similarity in monoallelic methylation patterns. Analyzing siblings allowed us to assess if genetically related individuals had more similarity in methylation patterns than unrelated individuals. At any given portion of the genome, two siblings can share zero, one or two of their parental chromosomes identical by descent (IBD). We defined all of these regions for each of 10 possible sibling pairs using the genome-wide genotyping data. Regions at which two siblings share no chromosomes IBD are only as similar in sequence as unrelated individuals from a similar genetic background. At the other end of the spectrum, regions in which two siblings share two chromosomes IBD are expected to have identical sequences (except for infrequent *de novo *mutations). We restricted the analyses to 5,660 amplicons that were homozygous in both parents and heterozygous in each of the five siblings. For each sibling pair, we then assigned each of the 5,660 heterozygous amplicons to genomic regions defined by the sharing of zero, one or two chromosomes IBD. Next, we determined the extent of methylation overlap in these regions and calculated the excess overlap above what would be expected by chance given the extent of monoallelic methylation in each individual. For all 10 sibling pair comparisons, the regions with two chromosomes shared IBD had the highest excess methylation overlap, followed by the regions with one chromosome IBD and then areas with zero chromosomes IBD. For nine of the 10 sibling pairs, the results were significant (*P *values ranging from 3 × 10^-3 ^to 1.7 × 10^-6 ^). The excess methylation overlap observed in the regions with two chromosomes shared IBD was around twice as frequent as in the unrelated individuals (see Additional file 7). Figure 1c shows data from the sibling analyses along with data from the analyses of unrelated individuals and parent-child analyses (see also see Additional file 8).

In addition to again showing a role for common polymorphisms in marking differences in local methylation, these analyses of sibling pairs highlighted an additional component of heritability most easily explained by the increased sequence identity in IBD regions. However, very local private polymorphism (within a few hundred base pairs) seems not to be a major factor because our sequencing experiments showed that beyond the known SNPs there were no other sequence changes for 20 tested amplicons. It is worth noting that inheritance of the methylation status itself could also contribute to the increased similarity of methylation patterns observed in the IBD chromosomes.

### DNA methylation in the immediate vicinity of SNPs in which one allele is part of a CpG dinucleotide

One way to assess the possibility that the sequences including and immediately surrounding the common SNP sites are themselves influencing local DNA methylation is to examine the relative positions of the SNPs (assayed by the array) and the MSRE sites on amplicons revealing methylation overlap. Skewing of the distribution of the distances between SNPs and MSRE sites towards shorter distances (than expected by chance) would provide evidence that the sequence surrounding the SNPs was directly involved, and would give some indication of the distance over which the effect on local methylation can be exerted. Note that the absence of skewing towards shorter distances would not rule out the possible involvement of the sequences surrounding the SNPs, but would merely indicate that if those sequences were directly involved, the distances over which they could act would be similar to or greater than the typical amplicon size.

To examine the relative positions of the MSRE sites and SNPs present on amplicons under analysis, we selected appropriate amplicons from the 5,660 that were informative in the sib-pair analyses. To focus on robust differences in the propensity for local methylation between the two alleles, we restricted these analyses to the 236 amplicons of the 5,660 for which ≥3 siblings had methylation overlap. Of these 236, we first focused on the 92 amplicons for which sequence analyses showed only a single MSRE site present on the amplicon, so as to allow unambiguous determination of the distance between the SNP and the examined MSRE site.

**Figure 2:**
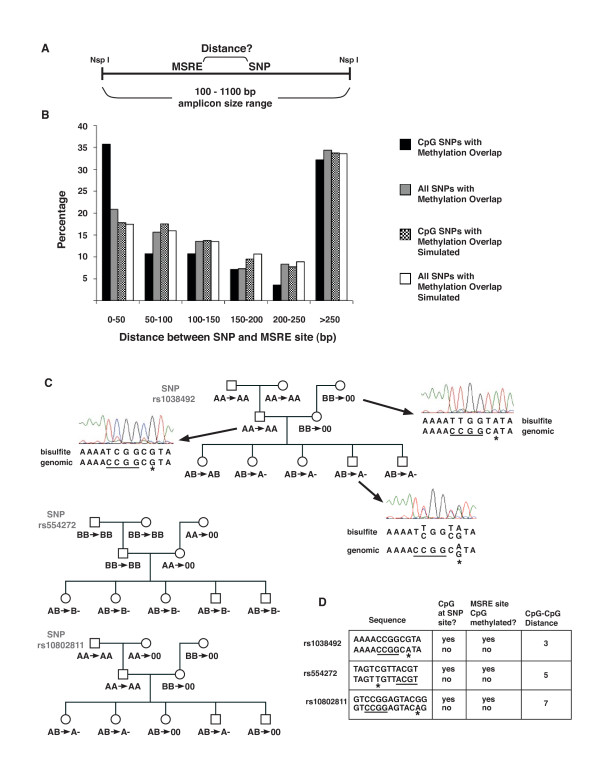
**The distribution of the distances between the single nucleotide polymorphisms (SNPs) and the methylation-sensitive restriction enzyme (MSRE) sites present on amplicons displaying methylation overlap**. **(a) **Generic amplicon with a single MSRE site. **(b) **Percentages of amplicons with given distances between the MSRE and the SNP, for amplicons with one MSRE site; 50 bp bins. See legend. **(c) **Allelic methylation patterns were analyzed in 10 individuals from three generations (grandparents, parents, children) using array-based methylation assessment (both genotyping and raw intensity assays), and bisulfite sequencing. For the three SNPs shown, the parents were homozygous for opposite alleles and all the children were heterozygous. Array-based genotyping data before and after MSRE treatment are separated by arrows. When the genotype call was homozygous after MSRE treatment, we denoted it as A or B. For the parents and grandparents, when the genotypes were all homozygous (for these SNPs) the genotype after MSRE treatment is displayed as AA or BB, reflecting the likelihood that both alleles were methylated, but the genotype calling would not itself reveal if one allele were unmethylated. Representative bisulfite sequencing results in one individual from each of three generations for the SNP rs1038492. The linked MSRE is underlined and the SNP is indicated with an asterisk. The 'A' allele is consistently methylated both at the SNP CpG and at the MSRE CpG. The 'B' allele is unmethylated at the MSRE site, so its genotype tends to disappear after MSRE treatment; upon bisulfite treatment, the 'B' allele has the C that is part of the CpG within the MSRE completely converted to T. **(d) **For SNPs in which one allele is a CpG, the SNP CpG was itself methylated along with the linked CpG within the MSRE.

For these 92 amplicons, although the distribution of the distances between the SNP and the MSRE site present on a given amplicon was essentially similar to the distribution from simulated random placement, a clear tendency towards closer proximity was observed for the subset of the overlapping amplicons in which one SNP allele forms a CpG ('CpG SNP'; Figure 2a, b). Of 28 such amplicons, 10 had a distance between the CpG SNP and the examined MSRE CpG of < 50 bp. We sequenced these 10 amplicons and found that the CpG SNP was the only difference between the two alleles. We further analyzed the methylation of these amplicons in more individuals from the three-generation pedigree and found a consistent although not uniform tendency for particular loci to be methylated on one of the two alleles in a larger pedigree (Figure 2c). Performing 1000 simulations with randomized placements of MSREs on the 28 amplicons, we found only 27 trials in which ≥10 (of 28) SNP-MSRE distances were < 50 bp (*P *= 0.027). These data suggest that for those SNPs in which one allele forms a CpG, there was an influence of the sequence surrounding and including the SNP on DNA methylation of very nearby (< 50 bp) sequences. Other SNPs might also directly affect methylation, but this effect would probably be over longer distances, as mentioned above.

Another indication that SNP CpGs (in the context of their surrounding sequences) do indeed affect methylation of the MSRE CpGs, would be if the allele with the CpG showed a bias towards either the methylated state or the unmethylated state for the MSRE CpG while the allele lacking the CpG showed bias toward the opposite state. The null hypothesis would be independence of the methylation state of the nearby MSRE and the allele containing the CpG. Analyzing the 10 cases in which the MSRE site and the CpG-containing SNP were within 50 bp (Figure 2d), we found eight cases in which the CpG allele was in *cis *with the methylated MSRE site (*P *(> 7) = 0.05). Moreover, we also analyzed the set of 236 amplicons (out of the 5,660 for which ≥3 siblings had evidence of methylation overlap, including amplicons with only one MSRE and those with more than one), and found that this larger set also displayed a tendency for the CpG alleles to be in *cis *with the methylated MSRE sites (51 of 71 sites; 72%; *P *< 10^-3^). This tendency was also seen in comparisons of unrelated individuals (66%, *P *< 10^-8^) and parent-child pairs (70%, *P *< 10^-13^). We concluded that the presence of the CpG at the SNP is positively correlated with the methylation of proximate CpGs residing within the MSRE sites.

### Simulation experiments to estimate the genome-wide fraction of SNPs associated with differential local methylation

The pairwise analyses supported the idea that for a fraction of amplicons harboring polymorphic sequences, the two alleles have a difference in methylation propensity. However because this propensity was not complete, it was difficult to glean from these pairwise analyses the degree of the difference between the two alleles. In addition, the overall frequency of regions showing methylation propensity differences between the two alleles was not clear. We therefore used simulations to explore various models, varying two parameters: the fraction of amplicons over the genome displaying differences in methylation propensity between the two alleles and the extent of difference in allelic methylation propensity (for those amplicons within the fraction defined in the first parameter). Simulated datasets comparable with the actual data obtained from DNA samples were analyzed, allowing ascertainment of the extent of methylation overlap and opposite overlap. In addition, the fraction of amplicons displaying overlap for groups of three and four individuals was determined. The measurements on the simulated datasets obtained under various models were then compared with the data obtained from the actual DNA samples.

**Figure 3:**
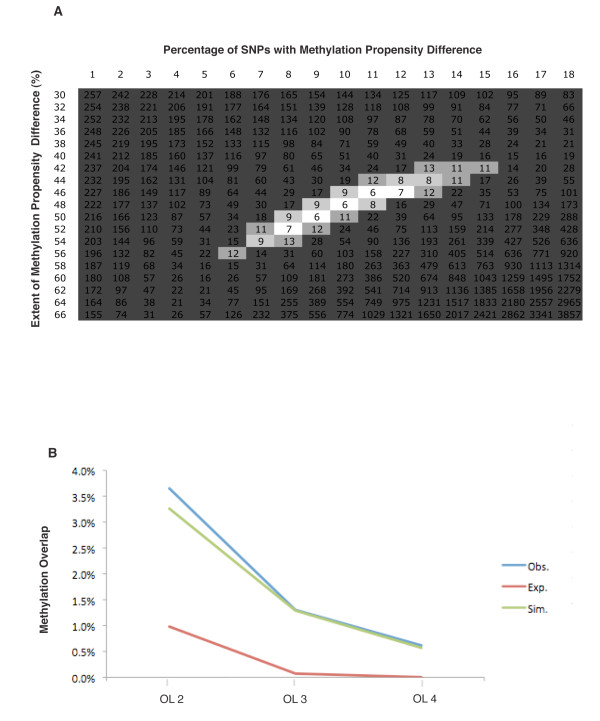
**Simulations allow estimation of the overall fraction of single nucleotide polymorphisms (SNPs) across the genome with differential local methylation**. **(a) **Under different simulations, we calculated the sum of squares of fractional deviations of the simulated data compared with empirical data examining fractional differences in four parameters: methylation overlap, opposite overlap and methylation overlap in three individuals, and methylation overlap in four individuals. **(b) **The fractions of amplicons showing methylation overlap in groups of two, three and four individuals for observed (Obs.), expected (Exp.) and simulated (Sim.) data.

Simulations that best approximated the observed data specified that ~10% of common SNPs were linked (or directly involved), with a difference in methylation propensity between the two alleles. Moreover, the extent of skewing was in the range of a 40% to 60% interallelic difference (Figure 3a). Results from one of the top scoring models are displayed in Figure 3b, along with the observed data and data that would be expected by chance; the percentages of amplicons displaying methylation overlap for groups of two, three and four individuals are presented. Note that in addition to providing a basis for comparison to the simulated data, the observed decay of the fraction of methylation overlap as increasing numbers of individuals are analyzed is informative: the fact that the observed methylation overlap fraction in groups of three or four individuals remained well above the values expected by chance is consistent with the idea that a substantial fraction of amplicons have some difference in methylation propensity between the two alleles (estimated by the simulations to be around 10%). Moreover, the decay demonstrates that the fraction of amplicons with an absolute or near-absolute difference in methylation propensity between the two alleles was small.

To examine other CpGs near to the ones assessed by the array-based experiment, we used bisulfite sequencing [10]. In addition to their usefulness in providing single-locus validation of the methylation results obtained from the arrays, these bisulfite sequencing experiments also allowed us to examine other CpGs that are not within MSRE recognition sites. Examining 30 non-MSRE CpGs on six amplicons displaying methylation overlap, we found that 23 had both alleles methylated, two had both alleles unmethylated and five had monoallelic methylation (see Additional file 9). These results are in line with the general CpG methylation rate across the genome (for CpGs outside of CpG islands). Notably, in each of the five cases in which nearby non-MSRE CpGs did show monoallelic methylation, it was in *cis *with the methylated CpG within the MSRE site. This is consistent with the idea that there is a local influence on methylation propensity of the sequence that includes the SNP, and that this influence can affect more than one CpG while not affecting other nearby CpGs, which remain predominantly methylated on both alleles.

## Conclusions

We analyzed 110,883 distinct small regions of the human genome for DNA methylation differences between the two alleles. Our data suggest that for ~10% of these regions there was a difference in the propensity for DNA methylation between the two alleles. We suggest the term 'sequence-influenced methylation polymorphism' (SIMP) to reflect this effect of sequence on local methylation propensity and to account for the fact that although the two alleles (defined by sequence) may have different propensities for methylation, it is not an all or nothing situation. The widespread nature of this type of epigenetic polymorphism would be expected to produce numerous chances for positional overlap with regulatory elements in the genome. Hence, in addition to the other ways mutations influence phenotype, these data suggest that sequence differences can have an effect on local DNA methylation, which in turn may affect the phenotype.

Owing to their high mutagenesis rate, the SNPs residing in CpG dinucleotides have been the focus of much attention in human genetics [11]. Our results further highlight the unique position of this large subgroup of SNPs in genetic-epigenetic interactions. Although they were not over-represented among amplicons displaying methylation overlap (considering their high frequency among all SNPs), their tendency to act from shorter distances and to have local methylation in *cis *with the CpG allele are intriguing properties because they suggest cooperativity of local methylation patterning during development and aging. The potential for cooperativity is interesting in the context of crystallographic data on the methyltransferase complex containing Dnmt3a/Dnmt3L which revealed a propensity for the simultaneous methylation of two nearby CpGs [12]. Given the known effect of methylation on CpG mutation rate [13,14], the observed effects of CpG SNPs on local methylation immediately suggests a model in which genetic change at one location can influence subsequent nearby genetic change through the epigenetic marker of DNA methylation.

## Methods

### Genomic DNA samples

Epstein-Barr virus (EBV)-transformed B-lymphocyte cell lines obtained from the National Institute of General Medical Sciences (NIGMS) cell repository http://ccr.coriell.org/nigms and cultured in RPMI-1640 medium with 15% fetal calf serum (FCS) (Sigma, St Louis, MO, USA) at 37°C and 5% CO_2_. Genomic DNA was purified (Mini Blood Kit; Qiagen, Valencia, CA, USA) according to the manufacturer's instructions. Ten samples from pedigree 1332: GM12096 (paternal grandfather); GM12097 (paternal grandmother); GM12099 (maternal grandmother); GM10848 (father); GM10849 (mother); GM12089 (daughter); GM12090 (son); GM12093 (daughter); GM12094 (son); GM12095 (son), and three samples from pedigree 45: GM12698 (father); GM12699 (mother); GM12706 (son) were analyzed. Peripheral white blood cell genomic DNA samples [PWBC1 (mother); PWBC2 (daughter) and PWBC3 (unrelated individual)] were obtained from J. Smoller (Massachusetts General Hospital, Boston, MA, USA). Postmortem genomic DNA samples were obtained from the Biochain Institute (Hayward, CA, USA).

### High-throughput methylation assay

Genomic DNA was genotyped on the 500 K SNP mapping arrays according to the manufacturer's instructions (Affymetrix). For methylation analysis, 1 μg of genomic DNA was digested at 37°C for 16 hours with an MSRE cocktail comprising *Aci*I (20 U), *Bsa*HI (1.3 U), *Hha*I (2.5 U), *Hpa*II (2.5 U) and *Hpy*CH4IV (10 U) (New England Biolabs, Ipswich, MA, USA), in a 50 l reaction volume with 1% bovine serum albumin and 10% NEB buffer no. 4 (New England Biolabs). The DNA was then inactivated by heating for 20 minutes at 60°C, precipitated with ethanol, and resuspended in Tris-EDTA buffer at 50 ng/μl; the usual hybridization procedure was then performed without further modifications. After this pre-treatment, the DNA was put through the mapping array procedure and further digested with either the *Nsp *I or *Sty *I restriction enzyme. Fragments of 200 to 1,100 bp (containing the polymorphic sites to be assessed) were amplified by PCR, and the resulting amplicons were then labeled and hybridized to the array.

Understanding that a genome-scale analysis of this type must guard against the potential for artifacts arising from polymorphisms in MSRE sites, we were careful to consider such a possibility at all stages of the project. In our previous analyses of the × chromosome, we used a filter that involved testing genomic DNA that had been pre-amplified with phi29 DNA polymerase (which generates an unmethylated representation of the genome) to ensure that the MSRE sites on both alleles were present. The phi29 DNA polymerase filter can deployed in the genome-scale analyses presented here, but we chose what turns out to be, practically, a more robust filter by excluding all SNPs on the Affymetrix array, which reside on amplicons containing any polymorphism in an MSRE site in the dbSNP database http://www.ncbi.nlm.nih.gov/projects/SNP. This filter based on dbSNP was used in all the analyses presented below, and although we know that this filter is discarding SNPs that do not reside on amplicons with MSRE site polymorphisms in the individuals examined here, we chose to use it to ensure that the analyses of allele-specific methylation would be robust.

Specifically, filtering out of SNPs resident on amplicons with possible polymorphisms was performed as mentioned in the text. Any amplicon with a polymorphism in dbSNP (build 129) that either could remove or create a recognition site for any of the five MSREs was removed. Similarly, we removed all amplicons for which there was any polymorphism that could remove an *Nsp*I or *Sty*I site at the end of the amplicon or lead to creation of a new 'internal' site. These filters were used for all such polymorphisms present at any frequency in the population. In addition, the amplicons residing on the × chromosome were removed to remove the possibility of effects on DNA methylation from the × inactivation process.

### Data analysis

Raw-intensity hybridization outputs for each clone before and after MSRE treatment were genotyped using the Dynamic Model Mapping Analysis (GCOS/GDAS software package; Affymertrix) or, for raw intensity analysis, normalized and compared with a pool of 13 genomic DNA samples from pedigrees 1332 and 45 using the dChip copy number analysis tool http://www.dchip.org. For statistical analyses, the non-parametric χ^2 ^test was used. For sibling-pair analysis, SNPs for which one of the parents was heterozygous and the other was homozygous were used to locate all recombination breakpoints occurring in each of the five children: GM12089; GM12090; GM12093; GM12094 and GM12095. The genomes of the 10 possible sibling pairs were then divided into regions for which the two pair members shared no, one or two identical pieces of the parental chromosomes. Finally, IBD SNPs and IBS SNPs were located in these regions and analyzed for methylation similarities.

### Validation of the high throughput allele-specific methylation assay

*Nsp*I fragments containing the arrayed SNPs were amplified from genomic DNA digested with MSRE, using PCR primers flanking the *Nsp*I sites. Both strands of the PCR product were sequenced, and the SNP readouts were compared with the genotypes obtained from the arrays. To validate methylation status, genomic DNA was converted using a bisulfite kit (EpiTect Bisulfite Kit; Qiagen), and fragments flanking the MSRE sites were amplified with bisulfite-specific primers and sequenced.

### Computer simulations

Programs used for the simulations are available on request. Briefly, epigenotypes for 10,000 'SNPs' were generated for each of four 'individuals' under varying differences between the methylation propensities of the two alleles. The allele with higher propensity for methylation was considered 95% methylated (the results of the simulations did not vary greatly when this percentage was changed slightly up or down). The starting genotype for each of the 10,000 SNPs was AB, and the epigenotypes (after MSRE treatment) were generated by treating each allele independently. For example, the more methylated allele would contribute to the epigenotype 95% of the time and the less methylated allele would contribute 95% minus 20% of the time if the simulation was testing a difference in methylation propensity of 20% between the two alleles. These 10,000 SNPs were assumed to be a varying fraction of the total analyzed SNPs for each given simulation (because the other parameter in the simulations was the fraction of SNPs across the genome that display a difference in methylation propensity). For the SNPs not subject to a difference in propensity for local methylation, switches to a homozygous call were assumed to be at a rate of 10%. The four individuals were then analyzed in a similar way to which the analyses of actual individuals were performed. We assessed methylation overlap and opposite overlap in pairs, and methylation overlap in groups of three individuals and groups of four individuals. The sum of the squares of the fractional differences provided a metric for evaluating the different simulations.

## Competing interests

The authors declare that they have no competing interests.

## Authors' contributions

AH carried out the experimental manipulations of DNA. AH and AC were involved in the overall design and coordination of the study, the analyses of data and simulations, and the writing and approval of the manuscript.

## Supplementary Material

Additional file 1 **Table S1**. Monoallelic methylation levels in individuals. Click here for file

Additional file 2 **Fig. S1**. Percentage monoallelic methylation for individual 12089 by chromosome. Note that the greater extent to which the × chromosome revealed allele-specific methylation in the Hellman and Chess 2007 experiment was because in that experiment, we were analyzing multiple subclones from each individual in order to define the effects of the × inactivation process on monoallelic methylation. Click here for file

Additional file 3 **Table S2**. Bisulfite sequencing control experiments. Click here for file

Additional file 4 **Table S3**. Methylation overlap between different (freshly isolated) tissues of unrelated individuals. Click here for file

Additional file 5 **Fig. S2**. Monoallelic methylation is largely independent of parental imprinting.
**(a, b) **Allele-specific methylation that is dependent on the parent of origin is a known feature of parentally imprinted regions. To explore whether parental imprinting is a major contributor to the observed methylation overlap across the genome, we analyzed individuals from three generations. Because the hallmark of an imprinted mark is that the inequality between the two alleles has the same parent of origin (maternal or paternal) in each generation, a sign of imprinting in this experiment would be allele-specifically methylated alleles tending towards having the same parent of origin in the first and the second transmissions observable in three generations. The configurations of the genotypes shown in (a) and (b) are representative of several that can inform such an analysis. **(C) **Averages and standard deviations for the analyses presented individually in Table S4 (Additional file 6). We observed equal frequencies of the same parent of origin as of a switch in parent of origin. These analyses did not rule out the possibility that a subset of methylation overlap was due to parental imprinting. Indeed, our analyses of the H19/IGF2 differentially methylated region revealed clustering of monoallelically methylated SNPs, suggesting that some methylation overlap could be due to imprinting. However, across the genome, parental imprinting was not a major contributor. Click here for file

Additional file 6 **Table S4**. Excess of methylation overlap is not due parental imprinting. Click here for file

Additional file 7 **Table S5**. Sibling pair analyses. Click here for file

Additional file 8 **Table S6**. Numbers of informative SNPs and of SNPs showing methylation overlap or opposite overlap among fifteen parent-child pairs. Click here for file

Additional file 9 **Table S7**. Bisulfite sequencing analyses of nearby CpGs. Throughout, the data were consistent with the Affymetrix array data and with what is generally known about CpG methylation across the genome. The analyzed CpGs had a range of distances from the SNP up to 1,045 bp. The predominant pattern is methylation of both alleles, which was found for 26 sites (and three more that were part of a methylation-sensitive restriction enzyme (MSRE) site and therefore we left out of the count). Five sites were methylated on the same allele as the methylated allele reported by the Affymetrix array experiment (and also an additional two sites that were MSREs and again, not counted). Two sites were unmethylated on both alleles. There were no examples of allele-specific methylation on the opposite allele compared with the Affymetrix array experiment. Click here for file
