# Supplementary material for: Extensive sequence-influenced DNA methylation polymorphism in the human genome
Source: Epigenetics Chromatin. 2010 May 24;3:11. doi: 10.1186/1756-8935-3-11 (PMC2893533; doi:10.1186/1756-8935-3-11)
Supplement: Additional file 1 — Table S1. Monoallelic methylation levels in individuals. [file 1756-8935-3-11-S1.PDF]

Table S1, Monoallelic methylation levels in individuals.

Generation	GM#	Gender	Age	Informative SNPs	AB	A-	B-	NoCall	%MAM
**Grandparents**	12096	M	94	23162	13752	1552	1367	6491	12.6
	12097	F	85	16542	7459	2216	1855	5001	24.6
	12099	F	73	26187	12780	2465	1963	8979	16.9
**Parents**	10848	M	48	25964	11436	2222	1792	10514	15.5
	10849	F	48	23748	12644	1689	1515	7900	13.5
	12698	M	na	22414	14638	1101	911	5768	9.0
	12699	F	na	21042	10365	2044	1664	6965	17.6
**Children**	12089	F	25	25247	12769	3046	2336	7096	21.3
	12090	M	22	25052	13268	2594	1896	7294	17.9
	12093	F	13	26915	10686	3770	2961	9498	25.0
	12094	M	8	24593	5851	4412	3831	10499	33.5
	12095	M	8	25821	9760	4032	3225	8804	28.1
	12706	M	na	28017	10694	3387	3231	10705	23.6
**Average**									**19.9**
**SD**									**6.9**
