# Supplementary material for: Extensive sequence-influenced DNA methylation polymorphism in the human genome
Source: Epigenetics Chromatin. 2010 May 24;3:11. doi: 10.1186/1756-8935-3-11 (PMC2893533; doi:10.1186/1756-8935-3-11)
Supplement: Additional file 2 — Fig. S1. Percentage monoallelic methylation for individual 12089 by chromosome. Note that the greater extent to which the × chromosome revealed allele-specific methylation in the Hellman and Chess 2007 experiment was because in that experiment, we were analyzing multiple subclones from each individual in order to define the effects of the × inactivation process on monoallelic methylation. [file 1756-8935-3-11-S2.PDF]

Figure S1, Percentage monoallelic methylation for individual 12089 by chromosome.

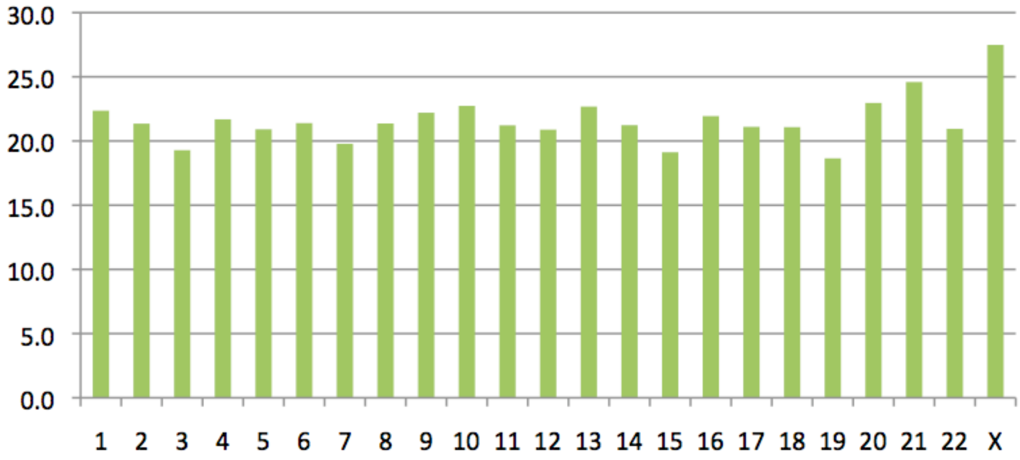

Note that the greater extent to which the X chromosome revealed allele-specific methylation in the Hellman and Chess 2007 experiment was due to the fact that in that experiment, we were analyzing multiple subclones from each individual in order to illuminate effects of the X-inactivation process on monoallelic methylation.
