# Supplementary material for: Extensive sequence-influenced DNA methylation polymorphism in the human genome
Source: Epigenetics Chromatin. 2010 May 24;3:11. doi: 10.1186/1756-8935-3-11 (PMC2893533; doi:10.1186/1756-8935-3-11)
Supplement: Additional file 3 — Table S2. Bisulfite sequencing control experiments. [file 1756-8935-3-11-S3.PDF]

Table S2, Bisulfite sequencing control experiments.

SNP	Forward bisulfite primer	Reverse bisulfite primer	Analyzed CpGs	Analyzed samples	Number of reads	Inconsisten-cies with array results
rs6615850	TGGTTTATTGTTTTATTGGGGAGT	TAATTCAAACCACCATCAACTCTTA	1	1	2	0
rs209235	GGTGTTTAAATTAAGTTGAATTAATGTATG	CCTACAAAAAATCCTAAAACCAATC	1	1	2	0
rs5955519	GGGGTTTGTAGATTTTTTTTGTAAG	AAACAATACCTACCACTCTTTCACTCT	1	1	2	0
rs16999756	GGGTTTTTGGGTGATAGATTTATTT	ACTCACCTTAACAATTTCCCATATATC	1	1	2	0
rs23963	ATAATGTTGTATTAGGGTTTAGTTA	CAAAAATTACCTACTCATATAAATC	1	1	2	0
rs2858769	TTTGGGATAAAAATTTTTGAAGTTTAA	CTTAAAAAAATTCTCTACTTTCAAAACAAA	1	1	2	0
rs10521584	TTGATGAAGATTTTGATGATAAAAA	AAATTAAAAAAAATAAATTCAAAAAAAA	1	1	2	0
rs10521584	TTAAGATAAAAAATTGAGGTTTTTT	ACATACCTAACCAATCATCACTA	1	1	2	0
rs4826507	CGAAGCTCCTTTGTTTCCTG	CCCCTGTACCCACTCACACT	2	4	8	0
rs5944690	GAGGGGTGTTGTTTGCATTT	TATGGTGAGCTGGCTTTTCC	2	4	8	0
rs17246798	AGTGTCATCCTCAGGCCAAT	GGTGCCTGTAATCCCAGCTA	2	4	8	0
rs1212068	CTGCCTCATGAACCCACTTT	TCCTTTCCTCCACCTCTGAA	2	3	6	0
rs2071211	TTTCTTCACGGCAGTTCTCA	AGTGGAGGTAGCAGGGGAGT	2	4	8	0
rs42890	CCTGGCTAACACGGTGAAAT	TTTTTGCCTTCAACCCTGTC	2	4	8	0
rs1038492	TTTGAAGATTAGGAGAATTTAAAGAGG	AAACACCAAATACCAACACCTTAAT	1	3	6	0
rs554272	TTTTGGTTTTAGTGAGTTGGAAAT	CAAACCTTTACCTTCAAAAATCTATC	1	3	6	0
rs2049623	GTTTAAAGATTAATAAGAAAATTTTAGTTG	AAAATAATAAAAAAAACCCAAAATAATAAC	1	3	6	0
rs10802811	TTGTAAGTAGATGATATGGAAAATATGTGA	AAAACCCAAATATCTATCAAAAAACC	1	3	6	0
rs4877289	TAGGTTAGATTGTAAGTTTTTGGAGAGTAG	ACAAATTTAATAAAAATAAAACCAATAAAA	1	3	6	0
