# Supplementary material for: Extensive sequence-influenced DNA methylation polymorphism in the human genome
Source: Epigenetics Chromatin. 2010 May 24;3:11. doi: 10.1186/1756-8935-3-11 (PMC2893533; doi:10.1186/1756-8935-3-11)
Supplement: Additional file 4 — Table S3. Methylation overlap between different (freshly isolated) tissues of unrelated individuals. [file 1756-8935-3-11-S4.PDF]

Table S3, Methylation overlap between different (freshly isolated) tissues of unrelated individuals.

Tissues compared	SNPs	OL-same	OL-opp.	p Value
Blood-Brain	5741	66	23	5.16E-06
Blood-Kidney	5385	78	13	9.50E-12
Blood-Lung	6053	58	9	2.14E-09
Brain-Kidney	5273	67	35	1.53E-03
Brain-Lung	7069	46	11	3.55E-06
Lung-Kidney	5370	61	10	1.42E-09

Blood = peripheral white blood cells, WBCs.
