# Supplementary material for: Extensive sequence-influenced DNA methylation polymorphism in the human genome
Source: Epigenetics Chromatin. 2010 May 24;3:11. doi: 10.1186/1756-8935-3-11 (PMC2893533; doi:10.1186/1756-8935-3-11)
Supplement: Additional file 5 — Fig. S2. Monoallelic methylation is largely independent of parental imprinting. (a, b) Allele-specific methylation that is dependent on the parent of origin is a known feature of parentally imprinted regions. To explore whether parental imprinting is a major contributor to the observed methylation overlap across the genome, we analyzed individuals from three generations. Because the hallmark of an imprinted mark is that the inequality between the two alleles has the same parent of origin (maternal or paternal) in each generation, a sign of imprinting in this experiment would be allele-specifically methylated alleles tending towards having the same parent of origin in the first and the second transmissions observable in three generations. The configurations of the genotypes shown in (a) and (b) are representative of several that can inform such an analysis. (C) Averages and standard deviations for the analyses presented individually in Table S4 (Additional file 6). We observed equal frequencies of the same parent of origin as of a switch in parent of origin. These analyses did not rule out the possibility that a subset of methylation overlap was due to parental imprinting. Indeed, our analyses of the H19/IGF2 differentially methylated region revealed clustering of monoallelically methylated SNPs, suggesting that some methylation overlap could be due to imprinting. However, across the genome, parental imprinting was not a major contributor. [file 1756-8935-3-11-S5.PDF]

Figure S2, Monoallelic methylation is largely independent of parental imprinting.

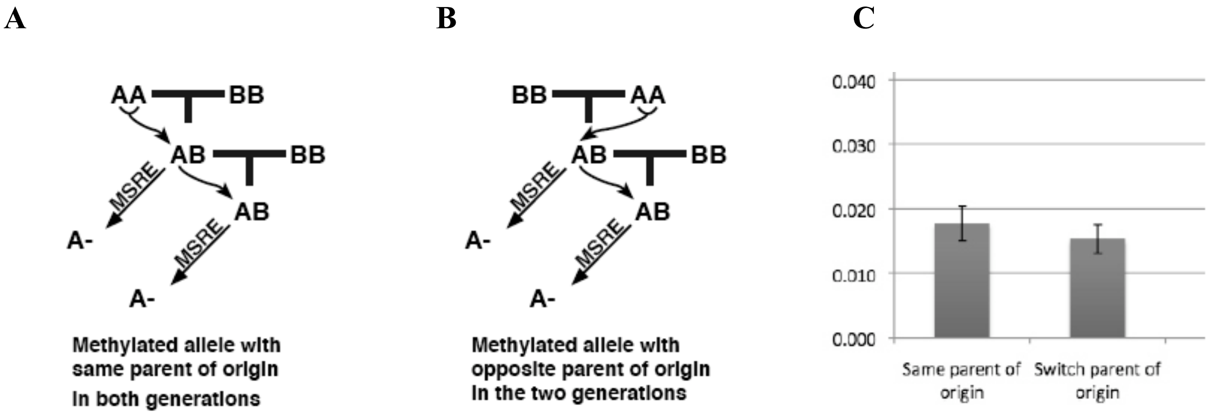

(A and B) Allele-specific methylation which is parent-of-origin dependent is a known feature of parentally imprinted regions. To explore whether parental imprinting is a major contributor to the observed methylation overlap across the genome, we analyzed individuals from three generations. Since the hallmark of an imprinted mark is that the inequality between the two alleles has the same parent-of-origin (maternal or paternal) in each generation, a sign of imprinting in this experiment would be that allele-specifically methylated alleles would tend towards having the same parent-of-origin in the first and the second transmissions observable in three generations. The configurations of the genotypes shown in panels A and B are representative of several that can inform such an analysis. (C) Displayed are the averages and standard deviations for the analyses presented individually Table S4. We observed equal frequencies of the same parent-of-origin as of a switch in parent-of-origin. These analyses did not rule out the possibility that a subset of methylation overlap was due to parental imprinting. Indeed, our analyses of the H19/IGF2 differentially methylated region revealed clustering of monoallelically methylated SNPs, suggesting that some methylation overlap could be due to imprinting. However, across the genome, parental imprinting was not a major contributor.
