# Supplementary material for: Extensive sequence-influenced DNA methylation polymorphism in the human genome
Source: Epigenetics Chromatin. 2010 May 24;3:11. doi: 10.1186/1756-8935-3-11 (PMC2893533; doi:10.1186/1756-8935-3-11)
Supplement: Additional file 6 — Table S4. Excess of methylation overlap is not due parental imprinting. [file 1756-8935-3-11-S6.PDF]

Table S4, Excess of methylation overlap is not due parental imprinting.

Parent	Child	Parent of Origin					
		Same			Switch		
		SNPs	Overlap	OL/Info.	SNPs	Overlap	OL/Info.
Father	GM12089	738	13	1.8%	2834	40	1.4%
Father	GM12090	1227	22	1.8%	1836	30	1.6%
Father	GM12093	1133	25	2.2%	2190	43	2.0%
Father	GM12094	996	19	1.9%	2324	53	2.3%
Father	GM12095	933	25	2.7%	2521	42	1.7%
Mother	GM12089	806	12	1.5%	2391	32	1.3%
Mother	GM12090	805	13	1.6%	2314	31	1.3%
Mother	GM12093	1163	16	1.4%	1942	24	1.2%
Mother	GM12094	1053	18	1.7%	1702	21	1.2%
Mother	GM12095	864	10	1.2%	2316	28	1.2%
Average				1.8%			1.5%
