# Supplementary material for: Extensive sequence-influenced DNA methylation polymorphism in the human genome
Source: Epigenetics Chromatin. 2010 May 24;3:11. doi: 10.1186/1756-8935-3-11 (PMC2893533; doi:10.1186/1756-8935-3-11)
Supplement: Additional file 7 — Table S5. Sibling pair analyses. [file 1756-8935-3-11-S7.PDF]

Table S5, Sib-pair analyses.

Pair	0 Chromosomes shared			1 Chromosome Shared			2 Chromosomes shared		
	SNPs	OL Freq	OL Freq Excess	SNPs	OL Freq	OL Freq Excess	SNPs	OL Freq	OL Freq Excess
**89-90**	1194	6.3%	3.2%	2667	6.4%	3.6%	1794	8.8%	5.8%
**89-93**	1287	7.1%	3.3%	2743	7.7%	3.8%	1625	9.4%	5.6%
**89-94**	1579	6.2%	1.5%	2426	7.6%	3.1%	1650	8.5%	3.8%
**89-95**	1559	7.2%	2.8%	2429	7.8%	3.8%	1667	9.5%	5.5%
**90-93**	1315	5.6%	2.3%	2586	7.1%	3.9%	1754	8.4%	5.3%
**90-94**	1215	5.3%	1.8%	2822	6.2%	2.3%	1618	8.3%	4.4%
**90-95**	1408	6.2%	2.8%	2541	6.7%	3.5%	1706	10.0%	6.2%
**93-94**	1238	7.0%	1.7%	3308	7.9%	2.9%	1109	9.6%	4.8%
**93-95**	1312	7.5%	3.1%	2959	9.0%	4.5%	1384	10.6%	6.0%
**94-95**	2247	8.1%	2.9%	2067	9.0%	3.3%	1341	9.5%	4.0%
**Average:**		6.7%	**2.5%**		7.5%	**3.5%**		9.3%	**5.1%**

OL Freq = Methylation overlap frequency
OL Freq Excess = Frequency in excess of that expected by chance
