# Supplementary material for: Extensive sequence-influenced DNA methylation polymorphism in the human genome
Source: Epigenetics Chromatin. 2010 May 24;3:11. doi: 10.1186/1756-8935-3-11 (PMC2893533; doi:10.1186/1756-8935-3-11)
Supplement: Additional file 8 — Table S6. Numbers of informative SNPs and of SNPs showing methylation overlap or opposite overlap among fifteen parent-child pairs. [file 1756-8935-3-11-S8.PDF]

Table S6, Numbers of informative SNPs, and of SNPs showing methylation overlap or opposite overlap among fifteen parent-child pairs.

arent	Child	Positions in pedigree	Pedigree	Informative SNPs	Parent A-	Parent B-	Child A-	Child B-	Observed Meth. OL	Observed Opp. OL	Expected Meth. OL	Expected Opp. OL	p value	(Obs.-Exp)/ Inf. Meth. OL
M12096	GM10848	Grandfather-Father	1332	10341	655	547	785	639	371	46	83.5	82.0	5.0E-57	2.8%
M12097	GM10848	Grandmother-Father	1332	7339	873	756	455	389	234	47	94.2	93.1	6.7E-29	1.9%
M12099	GM10849	Grandmother-Mother	1332	10521	855	718	699	625	411	52	99.5	98.5	1.7E-62	3.0%
M10848	GM12089	Father-Daughter	1332	11431	912	744	1328	1034	475	98	173.3	168.9	6.9E-56	2.6%
M10848	GM12090	Father-Son	1332	11610	947	745	1194	833	480	95	150.8	144.6	5.2E-58	2.8%
M10848	GM12093	Father-Daughter	1332	12128	1008	821	1684	1316	605	136	229.0	223.4	1.6E-66	3.1%
M10848	GM12094	Father-Son	1332	11217	912	743	1986	1745	587	160	277.1	273.4	5.1E-55	2.8%
M10848	GM12095	Father-Son	1332	11804	967	814	1806	1512	612	163	252.2	248.4	1.6E-58	3.0%
M10849	GM12089	Mother-Daughter	1332	10408	690	598	1169	927	343	90	130.8	128.6	5.2E-34	2.0%
M10849	GM12090	Mother-Son	1332	10335	674	611	1002	755	360	95	110.0	108.5	2.0E-35	2.4%
M10849	GM12093	Mother-Daughter	1332	10747	701	610	1434	1066	401	126	154.0	150.9	4.6E-33	2.3%
M10849	GM12094	Mother-Son	1332	10230	657	556	1738	1525	403	116	194.5	192.4	2.2E-36	2.0%
M10849	GM12095	Mother-Son	1332	10579	686	641	1560	1272	435	122	178.2	177.0	3.8E-40	2.4%
M12698	GM12706	Father-Son	45	10900	518	386	1266	1160	277	71	101.2	100.0	2.4E-28	1.6%
M12699	GM12706	Mother-Son	45	10388	944	762	1184	1136	509	134	190.9	190.1	1.7E-49	3.1%
