# Supplementary material for: Extensive sequence-influenced DNA methylation polymorphism in the human genome
Source: Epigenetics Chromatin. 2010 May 24;3:11. doi: 10.1186/1756-8935-3-11 (PMC2893533; doi:10.1186/1756-8935-3-11)
Supplement: Additional file 9 — Table S7. Bisulfite sequencing analyses of nearby CpGs. Throughout, the data were consistent with the Affymetrix array data and with what is generally known about CpG methylation across the genome. The analyzed CpGs had a range of distances from the SNP up to 1,045 bp. The predominant pattern is methylation of both alleles, which was found for 26 sites (and three more that were part of a methylation-sensitive restriction enzyme (MSRE) site and therefore we left out of the count). Five sites were methylated on the same allele as the methylated allele reported by the Affymetrix array experiment (and also an additional two sites that were MSREs and again, not counted). Two sites were unmethylated on both alleles. There were no examples of allele-specific methylation on the opposite allele compared with the Affymetrix array experiment. [file 1756-8935-3-11-S9.PDF]

Table S7, Bisulfite sequencing analyses of nearby CpGs.

SNP	SNP to MSRE CpG distance (bp)	Amplicon	Forward bisulfite PCR primer	Reverse bisulfite PCR primer	Distance from SNP (bp)	Allele-specific methylation from analyses of one AA and one BB individual
rs554272	5	I	TTGTATTTGAAGA GTATTGGGTTTG	ACAAATCTTATC CAAACAAAAAAA A	-169	M
		II	TGATAGATTTTTG AAGGTAAAGGTTT	CCCTTTTAACTCC ATTAATTAAATC TAAAT	199	M
					234	M
rs1080911 1	8	I	TAGTGTGTTTGGG TTGTTATAAAAA	CAAATAATAATA TAATAAATACTC CCAAAT	-242	M
		II	TAAAGAAATTAG GTATTTGGGAGTA TTTAT	ACAACATTAACT TCATAAAAAATT TAATCT	-83	M
					69	S
					130	U
rs1080281 1	7	I	GTGTATTTGAATT ATTTGGAAGGAA G	AAAAATCATTAT TTAACAACAAAA TTATTA	-7 (MSRE CpG)	S
					133	M
		II	TAAAGAAATTAG GTATTTGGGAGTA TTTAT	ACAACATTAACT TCATAAAAAATT TAATCT	169	S
					289	M
					347	M
					384	M
					464	S
rs6597434	3 MSRE sites	I	ATTTTTTTTAGGA TGTTAATAATTTT	CAAAAACTCCAA ATCTCCTTACTAT C	111	U
					116 (MSRE site)	M
					150 (MSRE site)	M
					156	M
		II	AAGGAGATTTGG AGTTTTTGAGTTT	ATTTTTAAATAA TTTTCCCTCTTAC C	382	M
					386	M
					388	M
					391	M
					402	S
					465	S
rs2182084	3 MSRE sites	I	GTATGAAGTTTTT TGGATTTTTTTTT	TCCCCTAACAAT AAACCATCAAC	-74 (MSRE site)	S
					84 (MSRE site)	M
					109	M
rs1083004 1	3 MSRE sites	I	TTTGTAGTTGTTTT TGGAGTTTTTA	CCTCATTAACCC AAAAATTCTA	-1045	M
					-1037	M
					-1029	M
					-919	M
					-917	M
					-911	M
					-878	M
		II	AATGAGGTTTTTG ATTTTTTTT	TCAAAATATAAA ATAATTTTTCTCT TTTAC	-565	M

(table S7 continued)

Key: S: same as in the array-based analysis
O: opposite to the array-based analysis
M: both alleles methylated
U: both alleles unmethylated

When there was only one MSRE site on the Nsp I amplicons used for the Affymetrix genotyping, then the distance is reported in the second column; otherwise the number of MSRE sites on that amplicon is reported. The column labeled "Amplicon" in this table refers to the PCR amplicons used for bisulfite sequencing in this experiment.

Throughout, the data are consistent with the Affymetrix array data and with what is generally known about CpG methylation across the genome. The analyzed CpGs had a range of distances from the SNP up to 1,045 base pairs. The predominant pattern is methylation of both alleles, which was found for twenty-six sites (and three more that were part of an MSRE site and therefore we leave out of the count). There are 5 sites that are methylated on the same allele as the methylated allele reported by the Affymetrix array experiment (and also an additional two sites that were MSREs and again, not counted). Two sites were unmethylated on both alleles. There were no examples where there was allele-specific methylation, but on the opposite allele as compared to the Affymetrix array experiment.
